# Rhabdomyoma and Hypoplastic Left Heart Syndrome - Case Report of a Very Rare Combination

**DOI:** 10.7759/cureus.19900

**Published:** 2021-11-25

**Authors:** Rahaf Waggass, Hanan S Bin Yahib, Hosam H Bin Seddeq, Aiman Shawli

**Affiliations:** 1 Cardiac Sciences, Ministry of National Guard - Health Affairs, Jeddah, SAU; 2 Medicine, King Saud bin Abdulaziz University for Health Sciences, Jeddah, SAU; 3 Medical Research, King Abdullah International Medical Research Center, Jeddah, SAU; 4 Pediatrics, King Saud bin Abdulaziz University for Health Sciences, Jeddah, SAU; 5 Pediatrics and Clinical Genetics, Ministry of National Guard - Health Affairs, Jeddah, SAU

**Keywords:** congenital heart disease, hypoplastic left heart syndrome, cardiac rhabdomyomas, tuberous sclerosis complex, echocardiography

## Abstract

The most benign cardiac tumor in the pediatric population is cardiac rhabdomyoma. They are known to be associated with tuberous sclerosis complex. Here we report a case with multiple cardiac rhabdomyomas and another rare anomaly of the heart known as hypoplastic left heart syndrome. The fetus was antenatally diagnosed with echocardiography which showed both rhabdomyoma and hypoplastic left heart. The patient was started on prostaglandin immediately after birth. He was confirmed postnatally to have inoperable congenital hypoplastic left heart syndrome. On the third day, the baby started to have progressive bradycardia and then died.

## Introduction

Rhabdomyomas can be categorized into cardiac and extracardiac tumors, which are considered rare mesenchymal tumors [1]. The majority of benign cardiac tumors are cardiac rhabdomyomas (CR), which are frequently diagnosed antenatally by echocardiography [2]. Out of all cardiac abnormalities that can be diagnosed prenatally by echocardiography, the diagnosis of rhabdomyoma accounts for nearly 1% [3]. It is commonly known that rhabdomyoma is associated with tuberous sclerosis complex (TSC) [4], an autosomal dominant genetic disorder. Rhabdomyomas caused as a result of mutations in either one of the protein complexes TSC1 (hamartin) or TSC2 (tuberin) [5]. Rhabdomyomas of the heart accounts for approximately 50% of patients with TSC, and 50% of patients with cardiac rhabdomyoma have TSC. The presence of more than one tumor highly suggests a diagnosis of TSC. In spite of the fact that it is rare to have vascular anomalies in TSC patients, some patients have coarctation of the aorta [6].

Another rare congenital heart disease is hypoplastic left heart syndrome (HLHS). It is an abnormal development of the left side of the heart that obstructs the left ventricle's blood flow. Moreover, the abnormality affects the development of the left ventricle, aorta, and arch of the aorta. This syndrome can also be accompanied by mitral atresia or stenosis. The degree of the blood flow obstruction, amount of involvement of the left side of the heart, and underdevelopment of both the aorta and left ventricle vary, causing a wide range of severity for this syndrome [7]. HLHS is usually associated with left ventricular outlet obstruction, and the severity of hypoplasia is mainly proportional to how much the outlet is obstructed [8]. It is considered a broad spectrum of congenital abnormalities of the heart; however, they all have a small left ventricle in common that cannot support systemic circulation. Most newborns with HLHS present with cardiovascular collapse, shock, weak peripheral pulse, and cyanosis at birth. Whenever HLHS is suspected, prostaglandin should be considered to maintain the patency of the ductus arteriosus, which in turn should prevent a lot of complications. In such cases, echocardiography is the modality of choice in providing the final diagnosis [9].

It is very uncommon to have a combination of these two congenital heart diseases. To our knowledge, there have been only two reported cases of patients having these two cardiac malformations together. In this report, we present another case diagnosed with multiple cardiac rhabdomyomas and HLHS, a complex congenital heart disease. In addition to atrial septal defect (ASD) and large patent ductus arteriosus (PDA) shown in the echocardiography. The first similar case was reported back in 1991, describing a patient with both CR and HLHS. Prenatal echocardiography was done for this patient that revealed a massive tumor in the left ventricle of the fetus's heart. The patient died of congestive heart failure 11 days after delivery. The patient's condition involved aortic atresia and underdevelopment of the ascending aorta [10]. The second reported case was in 2014, where they reported a case of HLHS that was secondary to CR. Fetal echocardiography at 25 weeks gestation revealed two huge masses in the left ventricle. One of them obstructed the left ventricle inflow; hence there was no outflow from the left ventricle to the aortic valve. The other mass was located in the ventricular septum below the aortic valve. Mild left ventricle hypoplasia, reduced left ventricular systolic function, and small mitral and aortic valves were noted in the echocardiography [11]. There is also a case of CR that was reported in 2014 in Saudi Arabia that appears relevant. This case did not have HLHS; however, mimicking it hemodynamically. The report described a massive left ventricular mass most likely to be rhabdomyoma supported with radiological evidence of tuberous sclerosis when brain computed tomography was done. In postnatal echocardiography, the mass occupied most of the left ventricular cavity, involved the mitral valve leaflets and chordae, and obstructed the left ventricular inflow and outflow as there was no flow across the mitral and aortic valves in colored Doppler study. There was a restrictive small ASD and a large PDA. The infant remained stable for 14 days; then, a repeated echocardiography in the third week showed a smaller tumor size than the previous study with some cavitation and some flow around it; however, the infant died after a week due to circulatory failure [12].

Studying this rare case and comparing it with the previously reported cases will help to gain a better understanding of future similar cases. It will also help to test the theories presented by previous studies regarding the connection between HLHS and rhabdomyomas.

## Case presentation

We present a case of a newborn male, the third child of a healthy Saudi consanguineous parent of a second degree, who had two normal siblings. The mother is 29 years old, Para three, and has one abortion with a history of rheumatic heart disease known to have hypothyroidism, and she is on thyroxine hormone. During pregnancy, echocardiography was done for her fetus at King Abdulaziz Medical City in Jeddah. The fetus was diagnosed as a case of cardiac rhabdomyoma and hypoplastic left heart syndrome. Multiple echogenic masses in the right ventricle without inflow and outflow obstruction, hypoplastic left heart, and hypoplastic ascending aorta were seen in the echocardiography. The neonate was a product of a spontaneous vaginal delivery at 38 weeks of gestational age with a birth weight of 3010 grams and appearance, pulse, grimace, activity, and respiration (APGAR) score of 8/1 and 9/5. The baby was admitted to the neonatal intensive care unit on his first day. Upon examination, the vital signs of the infant were as follows: heart rate 140-160 beats per minute, blood pressure 55-75/ 30-40 mmHg, respiratory rate 40-50 breaths per minute, and oxygen saturation 89-93 %. The baby was active and moving all his limbs. For the cardiovascular exam, he had normal heart sounds with a systolic murmur. Depending on the fetal echocardiography, the patient was started on prostaglandin infusion 0.05 mic/kg/hr immediately after birth for the patency of the ductus arteriosus.

**Figure 1:**
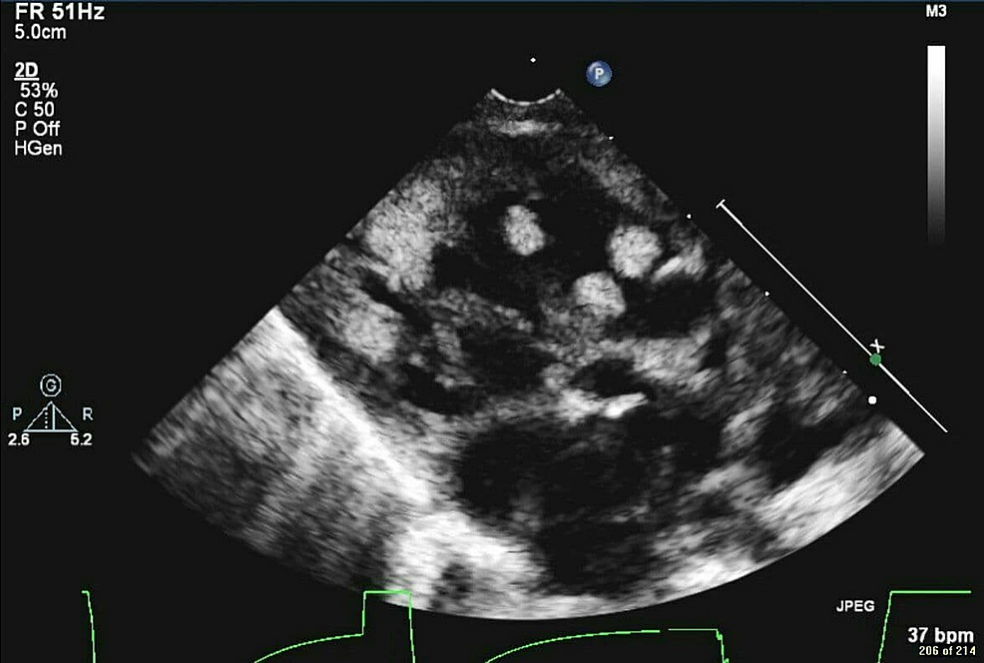
Echocardiography shows multiple echogenic masses (five at least seen in the ventricle and the largest is 9x8)

**Figure 2:**
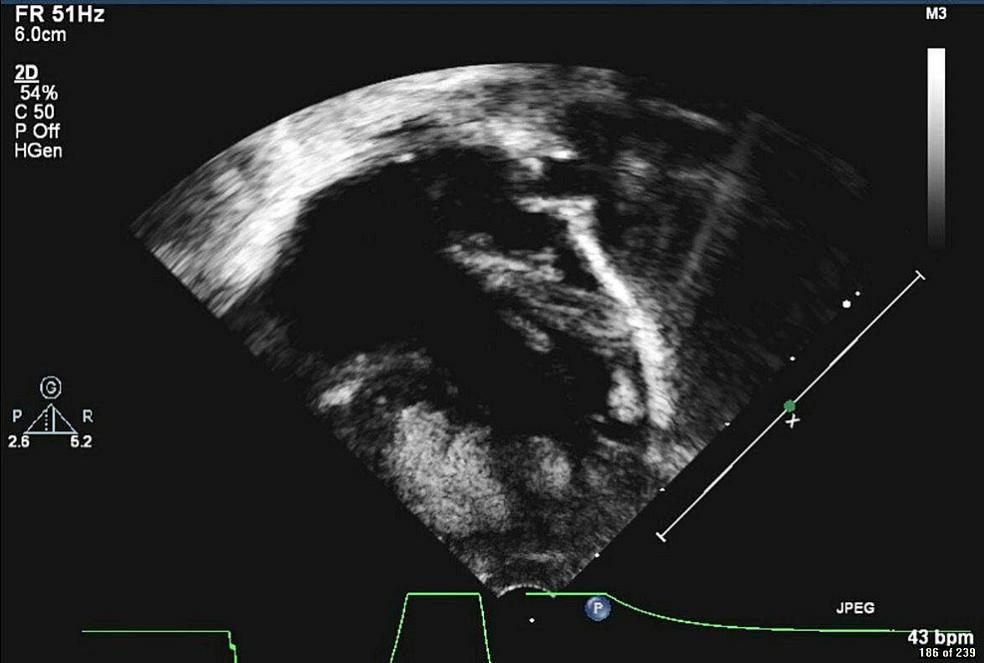
Echocardiography shows severe hypoplastic left ventricle

Following this baby's birth, echocardiography revealed levocardia, abdominal situs solitus, atrial situs solitus, S normal position of great vessels, and no pericardial effusion. Systemic and pulmonary venous drainage were both normal, and there was a moderate right atrial dilatation. Moreover, multiple echogenic masses (five at least) were seen in the ventricle, the largest one is 9x8 mm, and they were not causing any inflow or outflow obstruction (Figure 1). Echocardiography also showed severely hypoplastic left ventricle (LV), mitral valve atresia, aortic valve atresia, moderate tricuspid valve insufficiency, small aorta, and hypoplastic ascending and transverse aortic arch 1.9 mm (Figure 2). There was also a large patent ductus arteriosus, dilated main pulmonary artery with normal branch pulmonary arteries. Moreover, a patent foramen ovale (PFO) with a left to right shunt was seen. In terms of function, the patient had a normal single ventricular systolic function with only one atrioventricular valve. Overall, the echocardiography findings were suggestive of HLHS with CR. The patient was confirmed postnatally to have inoperable congenital HLHS. He had no dysmorphic features, he was neurologically fine, hypotensive with normal heart rate, but he had a systolic murmur. The genetic test revealed a heterozygous variant of uncertain significance in TS.

On the second day, an abdominal x-ray was done, and it did not show any obvious pathology. In addition, the prostaglandin was discontinued after a meeting that was done with the pediatric cardiologist, cardiac surgeon, cardiac intensivist, and neonatal intensivist, who all agreed on not proceeding since the patient had very complex anatomy. On the third day, the lab tests added that the patient had renal failure. Moreover, the baby started to have progressive bradycardia then asystole.

## Discussion

Wacker-Gussmann et al. reported an incidence of heart tumors in fetuses falls between 0.08% to 0.2% [13]. However, these percentages are thought to be underestimating the real numbers since there are some difficulties in recognizing the masses in the echocardiographic screening, and the regression of this type of tumor is common [14]. This is consistent with what was reported in previous research, where all the masses of CR were regressed after birth, at least partially in all the nine patients they studied [15]. Commonly CR involves the interventricular septum, or right ventricle [16], and another research noted the LV as a common site also [15]. In our case, multiple echogenic masses were recognized by the imaging in the right ventricle.

One of the several hypotheses of the development of HLHS is the interference with blood flow from the ventricles, which may lead to hypoplastic ventricles, and lack of outflow through the aortic valve may lead to underdevelopment of the ascending aorta, which emphasize that the location of CR is the most critical feature [11]. This case report demonstrates an example of a case with a combination of HLHS and CR with multiple masses but without any inflow or outflow obstruction due to CR. On the other hand, one of the previously reported cases with this rare combination had the HLHS as a complication to the CR due to an obstruction in the left side of the heart by one or more masses. It was a case reported in 2014 where the patient had two bulky masses, one obstructed the left atrioventricular valve, and the other was recognized in the interventricular septum under the aortic valve [11]. The other previous reported case was reported in 1991, with the same abnormalities, but the author did not specify whether the large mass in the LV caused an outflow or inflow obstruction or not [10].

CR can have a strong association with TSC, as Pietro Sciacca et al. reported some cases of CR associated with TSC where 93.9% of the patients he studied were diagnosed clinically with TSC, 9% confirmed the diagnosis with genetic tests, and 24.2% had a family history with this genetic disease [17]. On the other hand, Toshihiko Watanabe et al. pointed out that both the mother and her newborn did not show any manifestation of TSC [10]. In this study, TSC is possible in our patient since the genetic test revealed that the patient has a heterozygous variant of uncertain significance in TS and because of the supportive phenotype of the patient. In the literature, there were many diseases reported in fetuses diagnosed with CR, for instance, severe arrhythmia, tetralogy of Fallot (TOF), Ebstein anomaly, down syndrome, cleft palate, polycystic kidney disease, and clubfoot deformity [18]. In this study, heart murmur and renal failure were detected along with HLHS and CR. HLHS alone can be lethal without surgical treatment, and it is considered the reason behind 25 to 40 percent of the mortality in all cardiac diseases in neonates [19]. Moreover, morbidity and mortality can be risks in the case of hemodynamically significant CR [20].

## Conclusions

In conclusion, HLHS and CR are both rare heart disorders. These two disorders can be detected antenatally, and usually, the HLHS is known to be a result of a left ventricular outflow obstruction caused by CR. However, this study showed a case that has both HLHS and CR but without an outflow obstruction.
